# A Rare Cause of Upper Airway Obstruction in a Child

**DOI:** 10.1155/2017/2017265

**Published:** 2017-06-13

**Authors:** H. Ahmed, C. Ndiaye, M. W. Barry, Aliou Thiongane, A. Mbaye, Y. Zemene, I. C. Ndiaye

**Affiliations:** ^1^Department of Otolaryngology, Fann University Hospital, Cheikh Anta Diop University, Dakar, Senegal; ^2^Albert Royer Pediatric Hospital, Cheikh Anta Diop University, Dakar, Senegal; ^3^Department of Otolaryngology, Mekelle University, Mekelle, Ethiopia

## Abstract

Ventricular band cyst is a rare condition in children but can result in severe upper airway obstruction with laryngeal dyspnea or death. The diagnosis should be considered in any stridor in children with previous history of intubation or respiratory infections. We report a case of a 4-year-old girl, received in an array of severe respiratory distress, emergency endoscopy was done, and a large ventricular tape band cyst obstructing the air way was found. Complete excision was made, and postoperative prophylaxis tracheotomy was done. The postoperative course was uneventful with improvement of clinical and endoscopic signs.

## 1. Introduction

Ventricular band cyst is a rare laryngeal malformation, which can be life-threatening with severe obstructions. This is a surgical emergency which has variable clinical manifestations, presented mainly by stridor and respiratory distress. Diagnosis is based on clinical examination, but direct endoscopy plays a diagnostic as well as therapeutic role.

## 2. Case Presentation

This is a 4-year-old patient with no significant previous medical history. She was followed for 1 year for intermittent dyspnea, which became persistent and progressively worsened a week before admission. She was presented to the emergency room on 03.02.2015 for severe laryngeal dyspnea.

**Figure 1:**
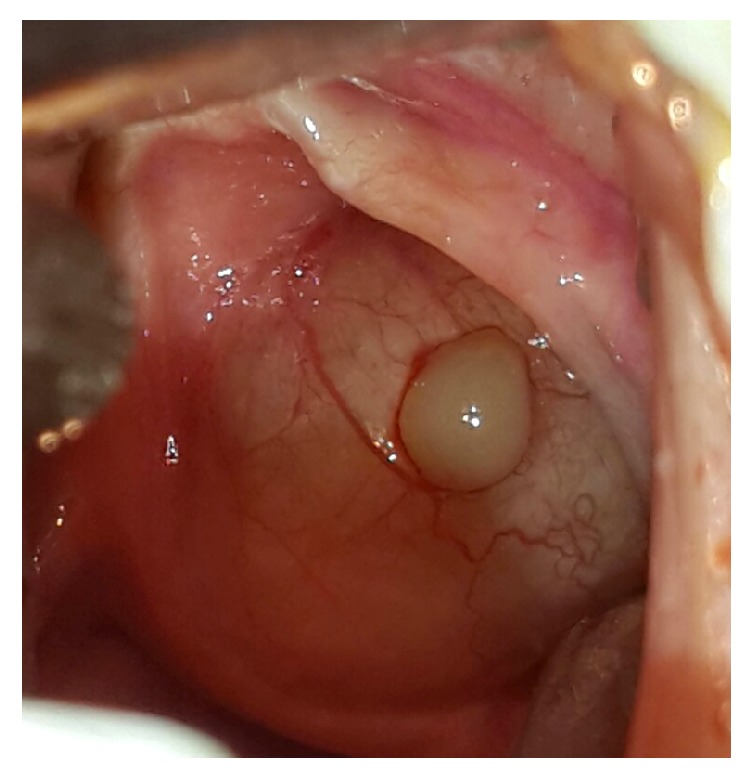
The cyst with its mucoid content after incision.

**Figure 2:**
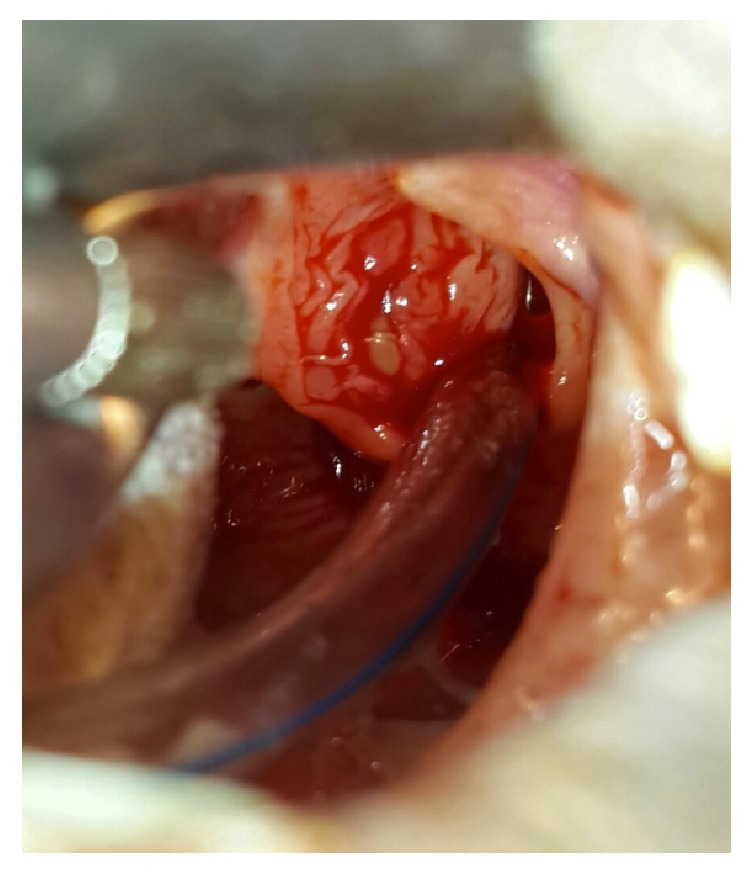
Cavity of the cyst after suctioning the mucoid content.

Clinical examination revealed an acute asphyxia with hyperextended neck and stage 4 laryngeal obstruction. Direct laryngoscopy was done urgently, and we found a round cyst, with vascular maze on the wall, originating in the left ventricle, encroaching on the root of the epiglottis, and completely blocking the vocal cords (Figure 1). The cyst was incised and thick mucoid fluid comes out, and marsupialization is done (Figure 2).

**Figure 3:**
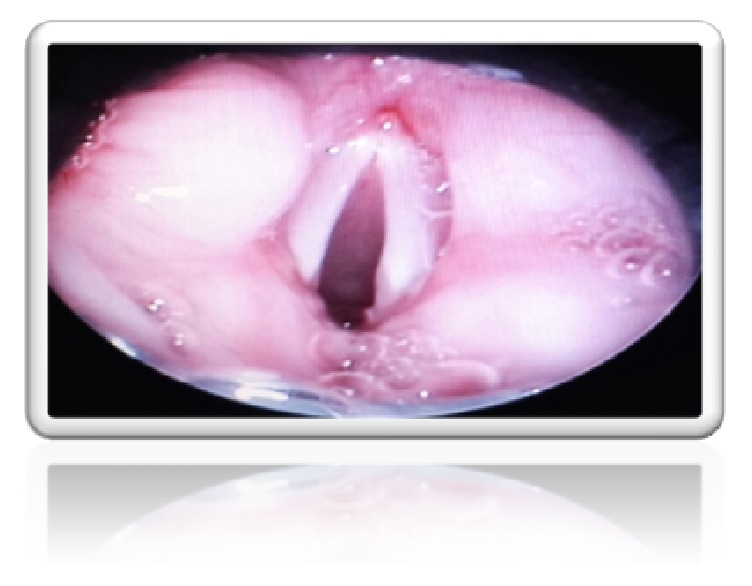
Control endoscopy.

**Figure 4:**
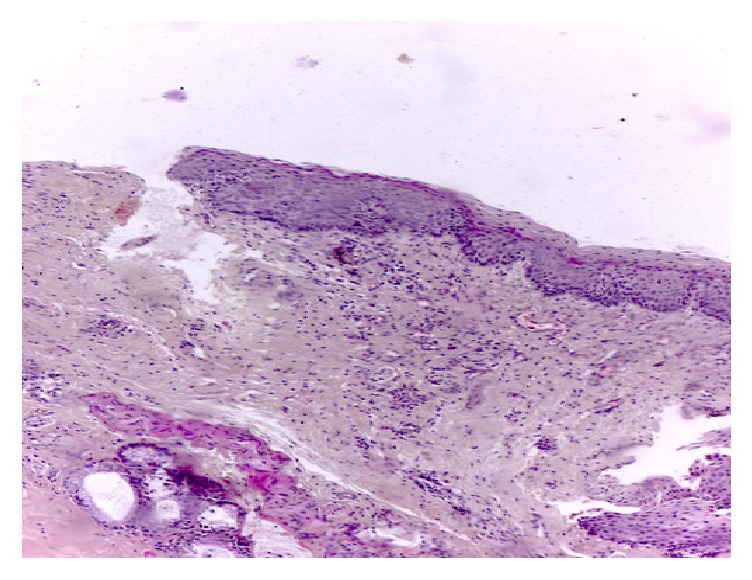
Histopathology finding of the ventricular band cyst.

Postoperative prophylaxis tracheotomy was performed. The postoperative course was uneventful. Postoperative control endoscopy was done at day 20 and a recurrence of the cyst was seen, for which complete resection was done endoscopically. The child is decannulated during this operation. A second control endoscopy was done at day 50 and the respiratory air way was free (Figure 3). The final histologic examination concluded a ductal ventricular band cyst (Figure 4).

It has been 20 months and there is no recurrence.

## 3. Discussion

Congenital or acquired laryngeal cysts are rare and are classified as glottic, supraglottic, and subglottic cysts. Zalagh et al. classified the laryngeal cysts according to their location: vocal cords (58.2%), ventricular fold (18,3%), vallecula (10.5%), epiglottis (10.1%), and aryepiglottic fold (2.2%) [1, 2]. There are two etiopathologic mechanisms for the development of cyst. The larger saccular cysts are congenital and are due to saccular atresia [3], while ductal cysts or retention cysts, more frequent, are acquired and result from obstruction of the mucous glands, by inflammation or trauma.

The epiglottic and ventricular band cysts are retention type [4]. Chronic inflammation leading to blockage of mucus glands is the main cause [5].

This phenomenon occurs especially during episodes of superinfections [6, 7] or laryngeal trauma and Mitchell et al. have described cases of laryngeal cyst in premature infants who underwent intubation in the first hours of their life [8].

We have not found a history of intubation in our patient but had episodes of respiratory infections, which could explain the formation of the cyst.

Clinical signs, depending on the size and location of the cyst, manifest as stridor, dysphagia, and respiratory distress due to the narrow laryngeal conduit in children [1, 2, 5, 8].

Such patients are often treated wrongly as asthma or laryngomalacia [7] as was the case in our patient.

Direct laryngoscopy remains the gold standard for the diagnosis of these laryngeal cysts [4, 5, 8–10]. The laryngeal ultrasound and CT scan can provide clarification, especially if there is a doubt with a laryngocele [9]. Typical laryngocele is filled with air and cyst is filled with mucus [11].

Treatment of these cysts is essentially surgical, endoscopic, or external approach. Excision of the cyst as a whole is the best method to prevent recurrence; [5, 12] the small cysts can be removed endoscopically, while the larger cysts require external approach [13]. Cases of laser vaporization have also been described [14].

In our case, the intervention had consisted of an incision with aspiration of the content and marsupialisation followed by prophylactic tracheotomy, giving priority to opening the airway of the patient. Tracheotomy may be used for intubation in large obstructive cysts [1, 13, 14].

## 4. Conclusion

Laryngeal cyst is a rare cause of laryngeal dyspnea but can be life-threatening because of its size and location. Endoscopy helps in diagnosis and treatment. Complete resection remains the treatment of choice. The frequency of recurrence requires regular follow-up.
